# Decoding foveal word recognition: the role of interhemispheric inhibition in bilateral hemispheric processing

**DOI:** 10.3389/fpsyg.2023.1293529

**Published:** 2023-11-30

**Authors:** Sangyub Kim, Kichun Nam

**Affiliations:** ^1^Wisdom Science Center, Korea University, Seoul, Republic of Korea; ^2^School of Psychology, Korea University, Seoul, Republic of Korea

**Keywords:** hemispheric coordination, interhemispheric inhibition, split fovea theory, bilateral projection theory, foveal word recognition

## Abstract

Extant research has largely favored the Split Fovea Theory (SFT) over the Bilateral Projection Theory (BPT) in the context of foveal word recognition. SFT posits that during foveal fixation, letters in the left and right visual fields are projected to their respective contralateral hemispheres, thereby facilitating a division of labor across the bilateral hemispheres. This division may serve as a regulatory mechanism to mitigate redundant processing in both hemispheres. The present investigation conducted two experiments utilizing Korean visual words to explore whether this hemispheric division in foveal word recognition is a strategy to circumvent potential interhemispheric inhibition arising from duplicated processing. Experiment 1 established the suitability of Korean visual words for studies involving both unilateral and bilateral presentations. Experiment 2 revealed that the split presentation of a word elicited greater accuracy compared to its identical presentation in the bilateral visual fields. These findings lend credence to the notion that interhemispheric inhibition may drive the hemispheres to engage in divided labor, thereby reducing processing redundancy in foveal word recognition.

## 1 Introduction

Building upon the foundational insights of Hellige (1993), the intricate interplay between the left and right cerebral hemispheres in cognitive functioning has been substantiated. Prior empirical investigations delineate that these hemispheres operate in a parallel yet autonomous fashion, each serving as a discrete computational entity (Iacoboni and Zaidel, 1996; Lindell et al., 2007). Within the neural architecture, a dynamically adaptive network—both functionally and structurally—facilitates a blend of concurrent and sequential processing modalities, thereby enhancing the computational efficiency of each hemisphere. Despite the myriad cognitive advantages conferred by this hemispheric specialization, an inherent regulatory mechanism within the inter-hemispheric interface fosters collaborative interactions. Specifically, inhibitory modulatory processes serve to integrate and harmonize the outputs emanating from each hemisphere, thereby precluding the emergence of potential computational discord between them.

Within the specialized domain of foveal vision as it pertains to visual word recognition, the academic landscape has been characterized by a dichotomy of theoretical paradigms (e.g., Ellis and Brysbaert, 2010). The first of these, the Split Fovea Theory (SFT), posits that the visual stimuli corresponding to the left segment of a word—based on the point of fixation—are selectively projected onto the right cerebral hemisphere, and conversely, the right segment is projected onto the left hemisphere. This segregated information subsequently undergoes interhemispheric transfer, primarily facilitated through neural conduits such as the corpus callosum (Brysbaert, 2004; Lavidor and Walsh, 2004). In contrast, the Bilateral Projection Theory (BPT) contends that foveally presented words are simultaneously propagated to both hemispheres, reserving contralateral projection exclusively for parafoveal words (Bunt et al., 1977). While both theories offer explanatory frameworks for the bilateral hemispheric mechanisms underlying foveal word recognition, recent empirical inquiries have increasingly lent credence to the SFT model (e.g., Brysbaert et al., 1996; Portin et al., 1998; Lavidor and Walsh, 2004; Hunter et al., 2007; Martin et al., 2007; Ellis and Brysbaert, 2010). These findings suggest a predilection of the bilateral hemispheres for segmenting foveal words and projecting them to their contralateral counterparts, as opposed to a simultaneous bilateral projection. This segmentation and subsequent contralateral projection, as posited by SFT, appear to confer computational efficiency, obviating the need for redundant processing across the hemispheres.

Boles (1990) examined a phenomenon of interhemispheric interruption when identical visual stimuli were presented in both the left and right visual fields. This observation intimates that an inhibitory mechanism operates between contralateral hemispheres during the visual recognition of bilaterally presented words, thereby casting doubt on the tenets of the BPT in the context of foveal word recognition. Given the brain’s proclivity for computational efficiency, such interhemispheric inhibition can be construed as an adaptive facet of hemispheric regulation. This adaptive mechanism serves to integrate, coordinate, and selectively curate outputs from each hemisphere, a process that is ostensibly essential for the harmonization of the bilateral neural system. Moreover, the empirical inclination toward the SFT in foveal word recognition may be predicated on the avoidance of computational redundancy across the hemispheres, engendered by duplicated projections. Such redundancy not only signifies inefficiency in hemispheric processing but also squanders valuable cognitive resources. Consequently, in the realm of foveal word recognition, the bilateral hemispheres appear to avoid interhemispheric inhibition, likely as a resource-conservation strategy, thereby aligning with the SFT framework wherein words are discretely segmented and projected to their respective contralateral hemispheres.

The present investigation employed a visual half-field presentation paradigm involving both split and identical word presentations to scrutinize the extent to which foveal word recognition aligns with the SFT as opposed to the BPT, particularly in the context of interhemispheric inhibitory regulation. In accordance with the visual half-field presentation paradigm, it is assumed that stimuli presented in the parafoveal region are initially processed by the contralateral hemisphere (Kim et al., 2020, 2022a,b, 2023; Kim and Nam, 2023a,b). Specifically, stimuli appearing in the right visual field (RVF) are initially processed by the left hemisphere (LH), and conversely, stimuli in the left visual field (LVF) are processed by the right hemisphere (RH). We posited the hypothesis that a split presentation of words in the left and right parafoveal visual fields would yield superior performance compared to the simultaneous presentation of identical words in those same fields. This split presentation is postulated to mirror the hemispheric division of labor, thereby aligning with the operational principles of the SFT. Intriguingly, the Korean language serves as an optimal linguistic medium for this line of inquiry, given its rigid syllabic boundaries characterized by either Consonant-Vowel-Consonant (CVC) or Consonant-Vowel (CV) structures, in contrast to the more fluid syllabic configurations found in many Western languages, including English. For instance, the Korean word “책상 (/chek-sang/)” is bifurcated into two distinct syllables, “책 (/chek/)” and “상 (/sang/),” in accordance with Korean’s stringent syllabic boundary rules. These syllables are then presented in contralateral visual fields, typically adhering to the left-to-right reading direction [“책 (/chek/)” in the LVF and “상 (/sang/)” in the RVF]. Consequently, the study also incorporated the separate presentation of word syllables to facilitate a comparative analysis with the simultaneous presentation of identical words across bilateral visual fields.

To rigorously interrogate the research hypothesis positing that foveal word visual processing aligns more closely with the SFT than with the BPT—primarily to circumvent interhemispheric inhibition due to redundant processing in the case of identical word projection to both hemispheres—two experiments were executed. The first experiment sought to ascertain whether the visual recognition of Korean words in the present study would also manifest the right visual field advantage (RVFA) and bilateral gain (BG) in laterally presented word recognition, consistent with extant literature. Previous investigations employing lateralized lexical decision tasks with Indo-European languages, notably English, have consistently reported RVFA, indicating superior recognition of words presented in the RVF as opposed to the LVF (Young et al., 1980; Bradshaw and Nettleton, 1983; Hellige, 1993; Mohr et al., 2007). Additionally, BG—defined as enhanced performance in bilaterally presented words relative to unilaterally presented words—has been consistently observed (Mohr et al., 2007). Experiment 1 corroborated the presence of both RVFA and BG in the context of a lateralized lexical decision task using Korean visual words, thereby establishing the suitability of Korean words for visual half-field studies.

In addition, Experiment 2 further delved into the comparative performance between split and identical word recognition in bilateral visual fields, utilizing Korean visual words as the experimental stimuli. We hypothesized that participants would manifest superior performance in split-word presentations relative to identical-word presentations within the bilateral visual field (BVF), a phenomenon attributed to hemispheric inhibitory regulation. To enable this comparative scrutiny, Experiment 2 utilized Korean visual words and assessed performance contrasting split and identical word presentations in the BVF. Furthermore, predicated on the split-fovea theory, we expected superior performance in the responses of split BVF presentations compared to those in the identical presentations in the BVF, and specifically to central visual field (CVF) if there is no corrupted effect from visual acuity, supporting the regulatory interaction between the two hemispheres due to avoid duplication of identical visual stimuli processing. In addition, if this regulatory interhemispheric interaction occurs before word representation stored in mental lexicon, then the benefits in split BVF presentation is observed in both words and pseudowords, meaning interhemispheric regulation in the early stage of visual word processing such as visual-perceptual processing stage. Otherwise, the benefits in split BVF presentation will be shown for words rather than for pseudowords, meaning emergence of regulatory interaction between the two hemispheres in the later stage of visual word processing after lexical access to words in mental lexicon. On the other hand, if the foveal word processing follows processing based on BPT, then we expected superior performance in responses of BVF presentations compared to those in split BVF presentations, meaning advantage from duplicated processing in the two hemisphere. And, likewise, if the benefits in BVF presentations occurs before word presentation, then it would show in both words and pseudowords, meaning the advantage from duplication in both hemispheres occurs irrespective of lexical access to mental lexicon. Otherwise, it would show only in words, meaning the advantage from duplication in both hemispheres only occurs when the stimuli are able to be accessed into mental lexicon.

## 2 Experiment 1

The primary objective of Experiment 1 was to assess the suitability of Korean visual words within a visual half-field presentation paradigm, focusing on the RVFA and BG. Initially, we posited that visual recognition would be compromised in parafoveal vision relative to foveal vision—a phenomenon termed the ‘visual acuity effect’—attributable to the increased viewing angle in parafoveal vision. Given that stimulus clarity generally diminishes with increasing distance from the point of fixation, we anticipated a decline in visual recognition irrespective of the lexicality of the stimulus. Furthermore, we hypothesized that if Korean words are indeed compatible with the visual half-field paradigm, they should exhibit a significant RVFA in parafoveal lexical decision, showing faster and/or more accurate responses for RVF presentation than LVF presentation in words in contrast with in pseudoword. This expectation is grounded in the notion that left-hemispheric dominance in language processing manifests as RVFA in lexical decisions for words as opposed to pseudowords (Knecht et al., 2000; Banich, 2003; Bourne, 2006). In addition, BVF words showed faster and/or more accurate responses for BVF presentation than for RVF presentation in words in contrast with in pseudowords, meaning significant BG only for words. This expectation is grounded in the notion that the co-activation of the bilateral hemisphere in cortical processing by simultaneous parafoveal presentation using identical words is evidenced by BG in lexical decisions for words relative to pseudowords (Hebb, 1949; Mohr et al., 2007).

### 2.1 Method

#### 2.1.1 Participants

In Experiment 1, a total of 25 participants, all native speakers of Korean, were recruited. The final dataset included all participants, as each adhered to the experimental protocol without exception, yielding a dataset devoid of missing responses or outliers. However, one participant, who registered a score of less than zero on the Edinburgh Handedness Inventory (Oldfield, 1971), was excluded from the final analysis to control for hemispheric asymmetry in language processing based on handedness. The final analytic sample consisted of 13 males and 11 females, with an age distribution of 23.96 ± 2.66 years (M ± SD). Handedness was rigorously controlled, as evidenced by scores on the Edinburgh Handedness Inventory (8.54 ± 1.35). All participants were confirmed to have no visual impairments in either eye and no documented history of mental or physical disabilities. Ethical clearance for the study was obtained from the Institutional Review Board of Korea University, and the study was conducted in strict adherence to the ethical guidelines outlined in the 1964 Declaration of Helsinki. Informed consent was obtained from all participants after they were fully briefed on the study’s ethical considerations.

#### 2.1.2 Experimental task

In Experiment 1, participants engaged in a lateralized lexical decision task, wherein they were tasked with discerning whether presented visual letter strings constituted legitimate words or pseudowords. The pseudowords, while orthographically and phonologically valid, lacked semantic content. Stimuli were displayed in one of four visual fields: central (CVF), left (LVF), right (RVF), or both (BVF). The sequence of stimulus presentation was randomized, and participants registered their responses via keyboard input, specifically employing the slash (‘/’) key for words and the ‘z’ key for pseudowords. Responses were executed using the index finger of either the left or right hand, with the responding hand counterbalanced across participants. The overarching directive for participants was to render their judgments with both alacrity and precision.

#### 2.1.3 Experiment procedure

The experimental protocol commenced with the display of a fixation point centrally positioned on the screen for a duration of 2000 ms. Upon its disappearance, visual letter strings were presented in one of the designated visual fields—central, left, right, or bilateral—for a temporal window of 180 ms. Participants were then allotted a 2000 ms timeframe within which to categorize the visual letter strings as either words or pseudowords. Prior to embarking on the 400 main trials, which comprised an equal distribution of 200 words and 200 pseudowords, all participants completed 16 practice trials to familiarize themselves with the task. To obviate the potential for stimulus overlap across different visual fields, each stimulus was presented only once throughout the entire experiment, facilitated by the implementation of a Latin square design. In total, four distinct stimulus lists, each containing 200 words and 200 pseudowords, were generated via the Latin square design, with each participant being assigned to one such list.

#### 2.1.4 Apparatus

The experimental stimuli were displayed using an RGB-colored LG monitor situated within a controlled experimental chamber. To ensure a consistent viewing distance, participants were instructed to position their chins on a chin rest, thereby maintaining a fixed 65 cm distance between their nasion and the screen. Furthermore, the visual angles for stimulus presentation were carefully calibrated to fall within a horizontal range of 2° to 5° and a vertical range of 1.5°, in accordance with established guidelines (Ellis et al., 1988; Metusalem et al., 2016). Experimental parameters and stimulus delivery were managed using E-Prime 2.0 professional software (Psychology Software Tools, Inc., Pittsburgh, PA). Participant responses were captured via a keyboard strategically positioned in front of them for ease of data collection.

#### 2.1.5 Materials

In the current experiment, a total of 200 noun words and 200 pseudowords served as the experimental stimuli. For methodological consistency, only two-syllable words and pseudowords were incorporated into the stimulus set. The word stimuli were extracted from the Korean Sejong Corpus, specifically selecting words with a frequency threshold of 10 or higher. Conversely, the pseudowords were constructed by amalgamating syllables present in actual words but were deliberately configured to be undefined within the Korean Sejong Corpus. As a result, these pseudowords were both orthographically and phonologically valid, yet devoid of semantic content.

#### 2.1.6 Experimental conditions

In the experimental design, two primary conditions were manipulated: the visual field of stimulus presentation and lexicality. The visual field condition encompassed presentations in the CVF, RVF, LVF, and BVF, thereby enabling a comparative analysis of response patterns contingent upon the specific visual field in which stimuli were displayed. Lexicality, on the other hand, served as an experimental variable designed to investigate differential responses between legitimate words and pseudowords.

#### 2.1.7 Statistical analyses

In Experiment 1, we performed mixed-effects regression analyses utilizing R software to scrutinize (1) the impact of visual acuity on both RTs and ACC for words and pseudowords, (2) the RVFA on RTs and ACC in words and pseudowords, and (3) the BG on both RTs and ACC for words and pseudowords (R Core Team, 2012). Each analytical model was formulated to incorporate both fixed and random effects, thereby offering a holistic framework for empirical inquiry. Fixed effects encompassed variables of visual field (VF), lexicality, and their two-way interaction (VF × lexicality). The VF delineated into CVF and BVF for examination of the impact of visual acuity, LVF and RVF for examination of the RVFA, and RVF and BVF for examination of the BG. The lexicality delineated into categories of word and pseudoword. Random effects were integrated into the model to account for inter-participant and inter-item variability, thereby ensuring a methodologically rigorous and nuanced analysis. We reported standardized beta values (*β*), standard errors (*SE*), t statistic, and value of p in the mixed effects regressions for RTs and ACC. The mixed-effect regression models were executed in the R software utilizing the lmer function for RTs and glmer function for ACC.

### 2.2 Results

**Figure 1:**
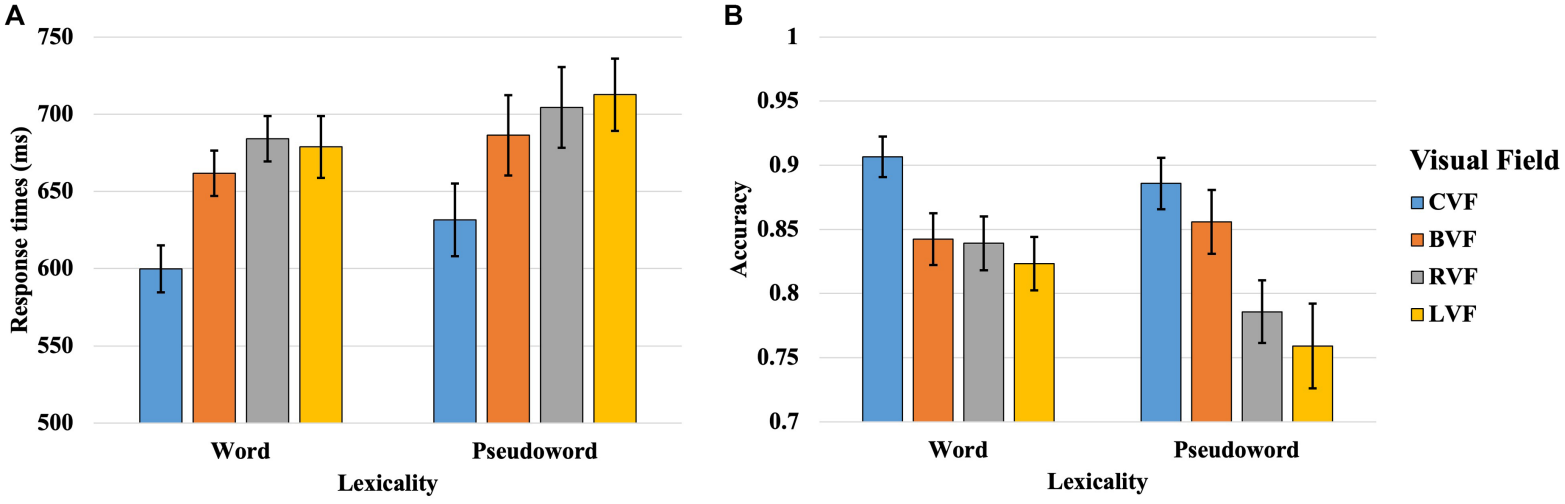
Results of the response times (**A**) and accuracy (**B**) in the CVF, BVF, RVF, and LVF in lateralized lexical decision task of Experiment 1. The line in the bar indicates standard error. The standard error is computed by dividing the standard deviation by the square root of the sample size. The standard error serves to assess how closely a statistic derived from the sample approximates the true parameters of the overall population.

Data were acquired for both response times (RTs) and accuracy (ACC) in the context of the lateralized lexical decision task. Preliminary analysis indicated that the ACC for both words and pseudowords across all participants fell within a range of three standard deviations, thus warranting the inclusion of all participant data in the final analysis. The outcomes pertaining to RTs and ACC are delineated in Table 1 and Figure 1, respectively.

**Table 1:** Results of the response times (RT) and accuracy rates (ACC) in the lateralized lexical decision task in Experiment 1.

	CVF		BVF		RVF		LVF	
	RT	ACC	RT	ACC	RT	AC	RT	AC
Words	600 (74)	0.907 (0.077)	662 (72)	0.843(0.099)	684 (73)	0.839(0.102)	679 (98)	0.823(0.102)
Pseudowords	632 (116)	0.886(0.098)	686 (128)	0.856(0.122)	704 (128)	0.786(0.119)	713 (115)	0.759(0.162)

The bracket value indicates the standard deviation.

#### 2.2.1 Investigation of visual acuity effect in the parafoveal lexical decision using BVF vs. CVF presentation

Initially, the outcomes for RTs revealed significant main effects for both VF [*β* = −0.119, *SE* = 0.009, *t* = −12.657, *p* < 0.001] and lexicality [*β* = 0.243, *SE* = 0.015, *t* = 16.338, *p* < 0.001]. However, the two-way interaction between VF and lexicality did not attain statistical significance [*β* = 0.006, *SE* = 0.009, *t* = 0.646, *p* = 0.518]. The significant main effect of VF suggested accelerated responses in the CVF compared to the BVF. Subsequent analyses of the VF main effect for both words and pseudowords revealed that the significant main effect of VF was attributable to both words [*β* = −0.141, *SE* = 0.014, *t* = −10.370, *p* < 0.001] and pseudowords [*β* = −0.109, *SE* = 0.013, *t* = −8.158, *p* < 0.001]. Moreover, the significant main effect of lexicality indicated expedited responses for words relative to pseudowords.

Subsequent to the RT analyses, the findings for ACC revealed significant main effects for both VF [*β* = 0.144, *SE* = 0.030, *z* = 4.786, *p* < 0.001] and lexicality [*β* = −0.216, *SE* = 0.057, *z* = −3.816, *p* < 0.001]. Nonetheless, the two-way interaction between VF and lexicality failed to reach statistical significance [*β* = −0.028, *SE* = 0.030, *z* = −0.931, *p* = 0.352]. The pronounced main effect of VF suggested enhanced ACC in the CVF as opposed to the BVF. Further dissection of the VF main effect for both lexical categories—words and pseudowords—indicated that the significant main effect of VF was attributable to both words [*β* = 0.178, *SE* = 0.046, *z* = 3.879, *p* < 0.001] and pseudowords [*β* = 0.120, *SE* = 0.040, *z* = 2.982, *p* = 0.003]. Moreover, the significant main effect of lexicality denoted superior ACC for words in comparison to pseudowords.

#### 2.2.2 Investigation of RVFA in the parafoveal lexical decision using LVF vs. RVF presentation

An initial analysis focused on RTs and the analysis yielded a significant main effect for lexicality [*β* = 0.244, *SE* = 0.015, *t* = 16.447, *p* < 0.001], as well as a noteworthy two-way interaction between VF and lexicality [*β* = 0.021, *SE* = 0.010, *t* = 2.231, *p* = 0.026]. In contrast, the main effect associated with VF did not attain statistical significance [*β* = −0.001, *SE* = 0.010, *t* = −0.100, *p* = 0.920]. The pronounced main effect for lexicality suggested more rapid RTs for words as compared to pseudowords. Subsequent exploration of the significant interaction between VF and lexicality through simple main effect analysis revealed that neither the effect of VF for words [*β* = −0.026, *SE* = 0.014, *t* = −1.902, *p* = 0.057] nor for pseudowords [*β* = 0.019, *SE* = 0.013, *t* = 1.402, *p* = 0.161] reached statistical significance.

Subsequent to the evaluation of RTs, the analysis was extended to examine ACC. The statistical output revealed significant main effects for both VF [*β* = 0.071, *SE* = 0.030, *z* = 2.363, *p* = 0.018] and lexicality [*β* = −0.215, *SE* = 0.056, *z* = −3.814, *p* < 0.001]. However, the interaction between VF and lexicality did not reach statistical significance [*β* = −0.051, *SE* = 0.030, *z* = −1.708, *p* = 0.088]. The main effect for VF suggested a heightened level of ACC in the RVF compared to the LVF. Upon disaggregating the VF effect by word and pseudoword categories, it was observed that the VF effect was primarily driven by words [*β* = 0.125, *SE* = 0.046, *z* = 2.735, *p* = 0.006], rather than pseudowords [*β* = 0.021, *SE* = 0.040, *z* = 0.527, *p* = 0.598]. Additionally, the main effect of lexicality indicated superior ACC for words relative to pseudowords.

#### 2.2.3 Investigation of BG in the parafoveal lexical decision using BVF vs. RVF presentation

Initial analyses were executed on RTs. The outcomes revealed salient main effects for both VF [*β* = 0.032, *SE* = 0.010, *t* = 3.355, *p* < 0.001] and lexicality [*β* = 0.243, *SE* = 0.015, *t* = 16.431, *p* < 0.001]. Contrarily, the two-way interaction between VF and lexicality did not attain statistical significance [*β* = −0.016, *SE* = 0.010, *t* = −1.721, *p* = 0.085]. The pronounced main effect for VF suggested expedited responses in the BVF compared to the RVF. Subsequent analyses partitioning the VF main effect by word and pseudoword categories revealed that the significant VF effect was attributable to words [*β* = 0.053, *SE* = 0.014, *t* = 3.841, *p* < 0.001], rather than pseudowords [*β* = 0.017, *SE* = 0.013, *t* = 1.280, *p* = 0.201]. Additionally, the main effect of lexicality indicated accelerated responses for words relative to pseudowords.

Subsequent to the RTs analysis, the findings for ACC revealed robust main effects for both VF [*β* = −0.120, *SE* = 0.030, *z* = −3.994, *p* < 0.001] and lexicality [*β* = −0.218, *SE* = 0.057, *z* = −3.849, *p* < 0.001]. In contrast, the two-way interaction between VF and lexicality did not reach statistical significance [*β* = 0.057, *SE* = 0.030, *z* = 1.882, *p* = 0.060]. The pronounced main effect for VF suggested enhanced ACC in the BVF as opposed to the RVF. Further dissection of the VF main effect by word and pseudoword categories indicated that the significant VF effect was attributable to words [*β* = −0.192, *SE* = 0.046, *z* = −4.168, *p* < 0.001], but not to pseudowords [*β* = −0.063, *SE* = 0.040, *z* = −1.561, *p* = 0.118]. Additionally, the main effect of lexicality underscored superior ACC for words relative to pseudowords.

### 2.3 Discussion

Experiment 1 aimed to examine the RVFA and BG in a lateralized lexical decision paradigm utilizing Korean visual words, in alignment with existing scholarly contributions (e.g., Mohr et al., 2007). Initially, the study revealed a pronounced visual acuity effect for both words and pseudowords, characterized by attenuated speed and ACC for parafoveal stimuli compared to foveal stimuli, irrespective of lexicality. This observation substantiates the notion of a decremental effect in parafoveal lexical decision-making, attributable to the increased viewing angle, thereby validating the visual half-field experimental design. Furthermore, the data corroborated significant RVFA and BG phenomena in the context of Korean visual word recognition, thereby replicating previous findings in other languages such as the RVFA in English (e.g., Barca et al., 2011), the BG in German (e.g., Mohr et al., 2007). The manifestation of RVFA implies a left-hemispheric predominance in the processing of Korean visual words (Knecht et al., 2000; Banich, 2003; Bourne, 2006), while the presence of BG suggests interhemispheric facilitation during bilateral word presentation (Mohr et al., 2007), in contrast to pseudoword conditions.

The presence of RVFA and BG in Korean, a language characterized by multisyllabic words, intimates that these phenomena are not contingent upon the morphological attributes of the words. This observation suggests the potential generalization of RVFA and BG in parafoveal word recognition across diverse linguistic architectures, including agglutinative (e.g., Korean) and alphabetic (e.g., English) languages. The consistency of RVFA and BG effects across languages suggests that language-specific traits, such as morphological structure, do not exert a significant influence on parafoveal word recognition. This universality underscores the left-hemispheric dominance and bilateral hemispheric cooperation in language processing, thereby affirming the methodological aptness of employing Korean visual words, particularly in parafoveal presentations, for future inquiries into hemispheric division of labor.

Furthermore, the strict syllabic demarcation inherent to Korean words offers a unique opportunity for subsequent experiments. Specifically, in Experiment 2, the use of Korean words will facilitate the exploration of interhemispheric inhibition through the manipulation of split-word presentations and the simultaneous display of identical words in the bilateral visual field. This is particularly pertinent for investigating interhemispheric inhibition predicated on the split-fovea theory, a manipulation that is more challenging to implement in languages like English, where syllabic boundaries are less rigidly defined.

## 3 Experiment 2

Experiment 2 aimed to investigate whether the foveal word recognition follows SFT rather than BPT due to a mechanism to mitigate interhemispheric inhibition arising from redundant processing during identical word presentations to contralateral hemispheres. We hypothesized that participants would manifest superior performance in split-word presentations relative to identical-word presentations within the BVF, a phenomenon attributed to hemispheric inhibitory regulation. To enable this comparative scrutiny, the experiment utilized Korean visual words and assessed performance contrasting split and identical word presentations in the BVF. Furthermore, predicated on the split-fovea theory, the study sought to compare the response of split BVF presentations with those in the CVF. This comparison was designed to discern whether the disparities between split and identical BVF presentation would endure when contrasting parafoveal split BVF processing with foveal central word processing. Should visual acuity effects persist in diminishing performance in split BVF processing, a notable divergence between split BVF and CVF lexical decisions is anticipated. Conversely, if split BVF processing aligns with the assumptions of SFT, irrespective of any decremental visual acuity effects, no significant difference between split BVF and CVF outcomes is expected.

### 3.1 Method

#### 3.1.1 Participants

In Experiment 2, an initial cohort of 43 native Korean speakers was recruited. One participant was subsequently excluded from the final data analysis due to non-compliance with experimental procedures, resulting in a final sample of 42 participants (15 males and 27 females; age 25.21 ± 4.03 years, M ± SD). Handedness was controlled across the sample, as evidenced by scores on the Edinburgh Handedness Inventory (8.19 ± 1.93) (Oldfield, 1971). All participants reported no visual impairments in either eye and had no documented history of mental or physical disabilities. Ethical approval for the study was granted by the Institutional Review Board of Korea University, Korea, where the research was conducted. The study was executed in strict compliance with the ethical guidelines stipulated in the 1964 Declaration of Helsinki. All participants were apprised of the ethical considerations and provided informed consent prior to their involvement.

#### 3.1.2 Experimental task

Experiment 2 also employed a lateralized lexical decision task, wherein participants were tasked with categorizing visual strings as either legitimate words or pseudowords. Notably, the pseudowords in this experiment were both orthographically valid and pronounceable, yet imbued with semantic content. Stimuli were displayed either in the CVF or the BVF. Within the BVF condition, two distinct types of presentations were utilized. The first entailed a simultaneous presentation of identical stimuli in the BVF; for instance, participants were exposed to the identical word ‘학교 (/hak-kyo/)’ in both the left and right visual fields concurrently. Conversely, the second type involved a split presentation in the BVF, wherein the word ‘학교 (/hak-kyo/)’ was bifurcated into its constituent syllables ‘학 (/hak/)’ and ‘교 (/kyo/)’, each of which was displayed separately in either the left or right visual field. The sequence of stimulus presentation was randomized, and participants registered their judgments via keyboard input, specifically employing the slash (‘/’) key for words and the ‘z’ key for pseudowords. Responses were executed using the index finger of either the left or right hand, with the responding hand counterbalanced across participants. The overarching directive for participants was to render their judgments with both alacrity and precision.

#### 3.1.3 Experimental procedure

**Figure 2:**
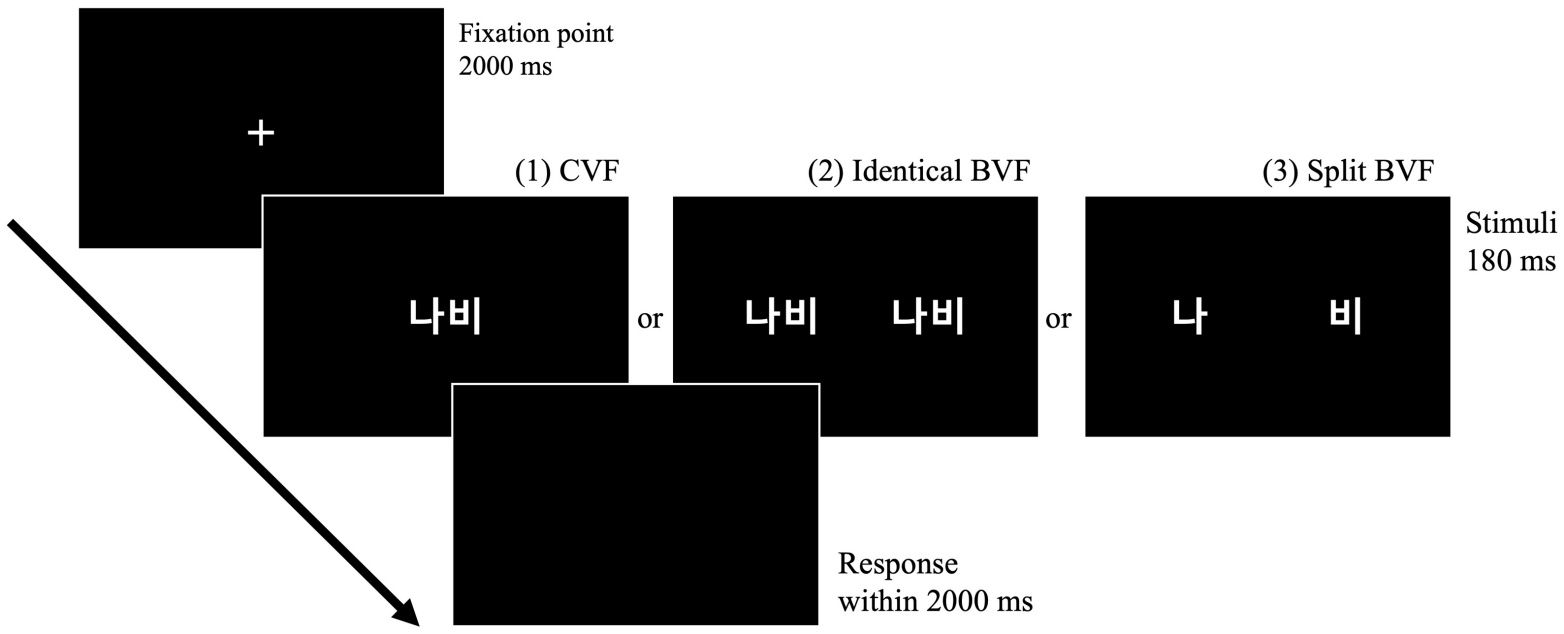
Schematic of the experimental paradigm employed in Experiment 2 for lateralized lexical decision task, illustrating stimulus presentation modalities in the CVF, identical bilateral visual field (identical BVF), and split bilateral visual field (split BVF).

The experimental protocol for Experiment 2 commenced with a centrally positioned fixation point displayed on the screen for a duration of 2000 ms. Subsequent to this, visual letter strings were presented either in the CVF or in one of two types of BVFs for a temporal window of 180 ms. Participants were allotted a 2000 ms timeframe within which to categorize these visual letter strings as either words or pseudowords. The complete procedural outline of Experiment 2 is delineated in Figure 2. Prior to embarking on the 396 main trials, which comprised an equal distribution of 198 words and 198 pseudowords, participants completed 12 practice trials for task familiarization. To mitigate the risk of stimulus overlap across different visual fields, each stimulus was presented only once throughout the experiment, facilitated by the implementation of a Latin square design. Consequently, three distinct stimulus lists, each containing 198 words and 198 pseudowords, were generated via the Latin square design, with each participant being assigned to one such list.

#### 3.1.4 Apparatus

Consistent with the methodology employed in Experiment 1, participants were subjected to the same experimental protocol.

#### 3.1.5 Materials

In Experiment 2, the stimulus set was derived from the materials utilized in Experiment 1, with the exclusion of two noun words and two pseudowords to align with the experimental design of the current study.

#### 3.1.6 Experimental conditions

In the experimental framework of Experiment 2, two primary conditions were manipulated: the visual field of stimulus presentation and lexicality. The visual field condition encompassed presentations in the CVF, as well as two types of presentations in the BVF—simultaneous and split. These variations facilitated a nuanced comparison of response patterns contingent upon the specific visual field in which stimuli were displayed. Lexicality served as an additional experimental variable, designed to examine differential responses between legitimate words and pseudowords.

#### 3.1.7 Statistical analyses

In Experiment 2, we performed mixed-effects regression analyses utilizing R software to scrutinize (1) the differential impact of split and identical BVFs on RTs and ACC in the context of words and pseudowords, and (2) the differential impact of split BVF and CVF on RTs and ACC across words and pseudowords (R Core Team, 2012). Each analytical model was formulated to incorporate both fixed and random effects, thereby offering a holistic framework for empirical inquiry. Fixed effects encompassed variables of visual field (VF), lexicality, and their two-way interaction (VF × lexicality). The VF delineated into split BVF and identical BVF for examination of the differential impact of split and identical BVFs, split BVF and CVF for examination of the differential impact of split BVF and CVF. The lexicality delineated into categories of word and pseudoword. Random effects were integrated into the model to account for inter-participant and inter-item variability, thereby ensuring a methodologically rigorous and nuanced analysis. The mixed-effect regression models were executed in the R software utilizing the lmer function for RTs and glmer function for ACC.

### 3.2 Results

**Figure 3:**
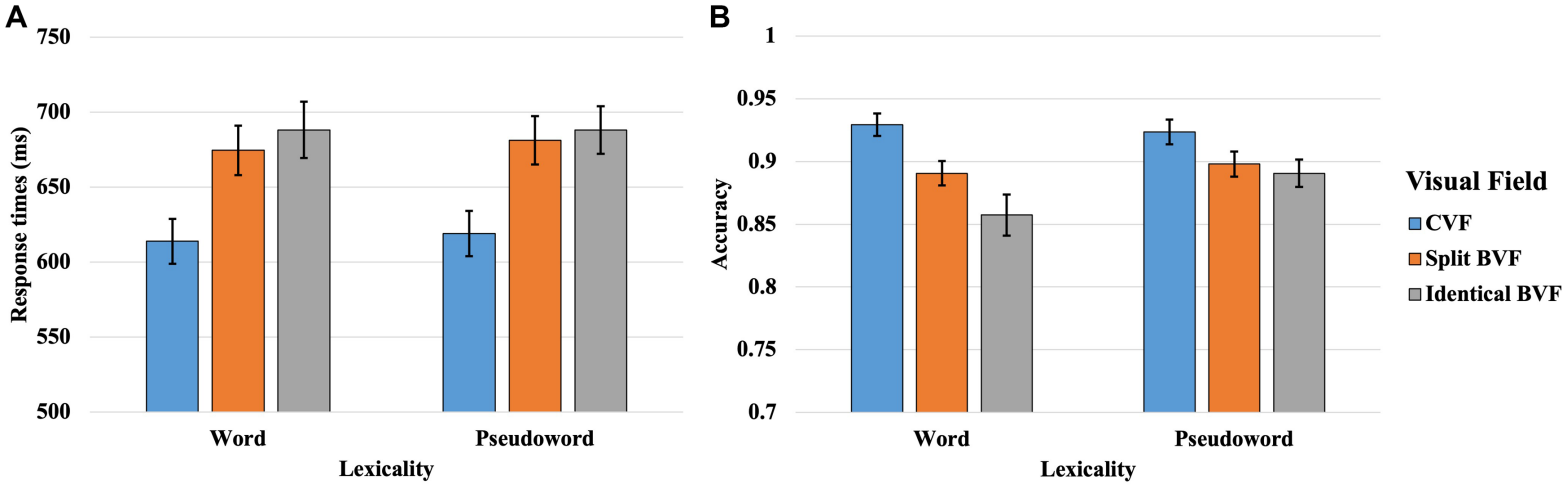
Results of the response times (**A**) and accuracy (**B**) in CVF, split BVF, and identical BVF in lateralized lexical decision task of Experiment 2. The line in the bar indicates standard error.

Data were amassed for both RTs and ACC in the context of a lateralized lexical decision task. Preliminary preprocessing analysis indicated that ACC metrics for both words and pseudowords were confined within a three-standard-deviation range for the entire participant pool, save for two outliers. To maintain the analytical robustness and integrity of the study, these two exceptional datasets were omitted from the final evaluation. The synthesized outcomes, delineated in Table 2 and Figure 3, expound upon the RT and ACC parameters observed in Experiment 2.

**Table 2:** Results of the response times (RT) and accuracy rates (ACC) in the lateralized lexical decision task in Experiment 2.

	CVF		Split BVF		Identical BVF	
	RT	ACC	RT	ACC	RT	ACC
Words	614 (96)	0.929(0.057)	675 (106)	0.891(0.063)	688 (121)	0.857(0.104)
Pseudowords	619 (97)	0.924(0.063)	681 (103)	0.898(0.064)	688 (102)	0.891(0.070)

The bracket value indicates the standard deviation.

#### 3.2.1 Investigation of SFT in lexical decision using split vs. identical BVF presentation

An initial analysis targeting RTs found statistically significant main effects for both VF [*β* = −0.022, *SE* = 0.007, *t* = −3.326, *p* < 0.001] and lexicality [*β* = 0.235, *SE* = 0.015, *t* = 15.828, *p* < 0.001]. Conversely, the interaction between VF and lexicality failed to reach statistical significance [*β* = −0.002, *SE* = 0.007, *t* = −0.371, *p* = 0.711]. The main effect of VF suggested accelerated RTs in the split BVF condition compared to the simultaneous BVF condition. Subsequent analysis of the VF main effect revealed that this acceleration was observed both for words [*β* = −0.021, *SE* = 0.009, *t* = −2.271, *p* = 0.023] and pseudowords [*β* = −0.024, *SE* = 0.010, *t* = −2.535, *p* = 0.011], indicating that the split BVF condition facilitated faster responses irrespective of stimulus lexicality. Additionally, the main effect of lexicality indicated a response time advantage for words over pseudowords.

Subsequent to the RT analysis, the analysis for ACC revealed statistically significant main effects for both VF [*β* = 0.066, *SE* = 0.029, *z* = 2.233, *p* = 0.026] and lexicality [*β* = −0.380, *SE* = 0.069, *z* = −5.513, *p* < 0.001]. Notably, a significant two-way interaction between VF and lexicality was also observed [*β* = 0.173, *SE* = 0.029, *z* = 5.862, *p* < 0.001]. The main effect of VF suggested enhanced ACC in the split BVF condition relative to the identical BVF condition. Concurrently, the main effect of lexicality indicated superior ACC for pseudowords compared to words. Further dissection of the significant VF × lexicality interaction revealed a significant simple main effect of VF for words [*β* = −0.130, *SE* = 0.047, *z* = −2.734, *p* = 0.006], signifying greater ACC in the split BVF condition. Likewise, a significant simple main effect of VF was found for pseudowords [*β* = 0.245, *SE* = 0.037, *z* = 6.680, *p* < 0.001], also indicating enhanced ACC in the split BVF condition.

#### 3.2.2 Investigation of visual acuity effect in the parafoveal lexical decision using split BVF vs. CVF presentation

An initial analysis of RTs yielded significant main effects for both VF [*β* = 0.136, *SE* = 0.007, *t* = 20.816, *p* < 0.001] and lexicality [*β* = 0.233, *SE* = 0.015, *t* = 15.616, *p* < 0.001]. Furthermore, a significant two-way interaction between VF and lexicality was observed [*β* = −0.018, *SE* = 0.007, *t* = −2.821, *p* = 0.005]. The main effect of VF revealed expedited responses in the CVF compared to the split BVF. Additionally, the main effect of lexicality indicated accelerated responses for words relative to pseudowords. Subsequent simple main effect analyses on the significant VF × lexicality interaction disclosed that the simple main effect of VF was significant for both words [*β* = 0.171, *SE* = 0.009, *t* = 18.657, *p* < 0.001] and pseudowords [*β* = 0.113, *SE* = 0.010, *t* = 11.910, *p* < 0.001], signifying more rapid responses in the CVF compared to the split BVF, irrespective of lexicality.

Subsequent to the RT analysis, the statistical outcomes for ACC revealed salient main effects for both VF [*β* = −0.238, *SE* = 0.031, *z* = −7.761, *p* < 0.001] and lexicality [*β* = −0.433, *SE* = 0.070, *z* = −6.210, *p* < 0.001]. Additionally, a significant two-way interaction between VF and lexicality was observed [*β* = 0.238, *SE* = 0.031, *z* = 7.775, *p* < 0.001]. The main effect of VF demonstrated enhanced ACC in the CVF as compared to the split BVF. Concurrently, the main effect of lexicality indicated superior ACC for pseudowords relative to words. A subsequent simple main effect analysis on the VF × lexicality interaction disclosed a significant simple main effect of VF for words [*β* = −0.498, *SE* = 0.052, *z* = −9.658, *p* < 0.001], signifying heightened ACC in the CVF over the split BVF. However, the simple main effect of VF for pseudowords was not statistically significant [*β* = 0.002, *SE* = 0.036, *z* = 0.065, *p* = 0.948], signifying no difference of ACC between the CVF and the split BVF presentations.

### 3.3 Discussion

Experiment 2 revealed that RTs were slower and ACC was diminished in the identical BVF as compared to the split BVF, irrespective of target lexicality. These findings suggest a superior visual recognition performance in the split BVF, lending empirical support to the split-fovea theory. Additionally, a significant performance discrepancy was observed between the split BVF and the CVF. This indicates that, despite the benefits of split presentation at parafoveal vision, a degradation in performance persists, attributable to the limitations of visual acuity in parafoveal presentations.

The findings of Experiment 2 corroborate extant literature, such as the work of Chiarello and Maxfield (1996), which posits the occurrence of interhemispheric inhibition during simultaneous presentation of identical words in both the left and right visual fields. This is evidenced by the slower RTs observed in the BVF in our study. Such inhibitory regulation between the hemispheres is postulated to serve as a compensatory mechanism aimed at mitigating redundant processing across both hemispheres. Given the brain’s proclivity for efficiency in cognitive processing, particularly in the context of mental energy conservation, interhemispheric inhibition serves to judiciously allocate limited neural resources. This shows the superior recognition performance for split words as compared to the simultaneous presentation of identical words in the BVFs.

An additional intriguing outcome of Experiment 2 was the absence of a significant delay in RTs for pseudoword processing in the BVF as compared to the split BVF, particularly when contrasted with word processing. This lack of delay in pseudoword processing suggests that interhemispheric inhibition in visual recognition is contingent upon lexical access to the mental lexicon, which is rather later stage of visual word processing such as lexical processing after visual-perceptual processing. This phenomenon can be attributed to hemispheric competition that arises during lexical access in the context of identical word recognition in the BVF. Such competition is engendered by the shared pathway for accessing the mental lexicon from both the left and right hemispheres.

The findings of this study lend empirical support to the SFT over the BPT in the context of foveal word recognition. The observed hemispheric conflicts, engendered by interhemispheric inhibition in BVF word recognition, suggest a predilection for SFT-based processing over BPT in foveal word recognition. When identical words are projected in the BVF, each contralateral hemisphere is activated to process the words via a shared lexical access pathway to the mental lexicon. This activation engenders hemispheric conflicts during lexical access, likely as a metabolic conservation strategy to mitigate the redundancy inherent in simultaneous activation of both hemispheres. In contrast, such conflicts are conspicuously absent in split BVF word recognition. In this condition, each hemisphere processes a distinct syllabic component of the word in the unilateral visual fields (UVFs), which are subsequently integrated to form a complete word. This obviates the need for redundant processing and the associated metabolic costs, thereby eliminating the delays observed in BVF word recognition. Thus, the superior performance in split BVF word recognition relative to BVF word recognition can be attributed to the mitigation of hemispheric conflict through interhemispheric inhibition, reinforcing the primacy of SFT in foveal word recognition.

## 4 General discussion

The present investigation conducted two experiments to ascertain whether foveal word recognition adheres more closely to the SFT than to the BPT, with a focus on the role of interhemispheric inhibitory regulation. Experiment 1 demonstrated the presence of RVFA and BG in a lateralized lexical decision task using Korean words. This outcome substantiates the feasibility of employing Korean words in visual half-field studies, akin to research conducted in other languages such as English. Experiment 2 revealed accelerated RTs in the visual recognition of split words presented in the BVF as compared to identical words also presented in the BVF. This finding suggests that the bilateral hemispheres engage in a division of labor to circumvent interhemispheric inhibition, particularly when identical words are propagated to both hemispheres, as opposed to split words in the BVF.

Indeed, interhemispheric inhibition serves as a critical regulatory mechanism for dynamic hemispheric processing within the brain. Chiarello and Maxfield (1996) delineated three distinct forms of interhemispheric inhibition. The first form entails functional suppression, wherein one hemisphere exerts inhibitory control over its contralateral counterpart during cognitive processing (e.g., Cook, 1984). Previous research elucidating this suppressive interaction posits that hemispheric dominance is achieved by one hemisphere inhibiting the other, thereby reducing parallel processing and mitigating potential conflicts between the hemispheres. The second form of inhibition is characterized by hemispheric isolation, aimed at alleviating potential bottlenecks in interhemispheric interactions (e.g., Zaidel et al., 1990; Hellige, 1993). This form is distinct from the first in that it allows for parallel processing within each hemisphere. Here, the interhemispheric transfer pathway is inhibited, effectively blocking communication between the hemispheres. While this blockade precludes interhemispheric interactions, it permits each hemisphere to function in parallel, thereby isolating them from each other. The third form of inhibition diverges from both functional suppression and hemispheric isolation, focusing instead on the restriction of one hemisphere’s efficiency by the other (Liederman, 1986; Clarke et al., 1993). This manifests as interhemispheric interference, wherein each hemisphere is presented with irrelevant or distracting information via the cortical pathways that facilitate interaction between the two hemispheres.

These three modalities of interhemispheric inhibition are posited to be instrumental in sustaining a harmonious and adaptive neural processing framework. Such inhibitory mechanisms between the hemispheres facilitate optimized hemispheric responses by mitigating redundancy. Given that duplicative processing across the left and right hemispheres is superfluous, it is plausible that one hemisphere exerts regulatory control over its contralateral counterpart to minimize redundant neural activations, particularly in the context of identical word projections to the BVFs.

This regulatory interplay between the left and right hemispheres may manifest as functional differentiation, potentially giving rise to hemispheric specialization—for instance, the left hemisphere’s dominance in language processing. Such regulatory mechanisms serve as a framework for the functional partitioning of tasks across the hemispheres. In this context, Karbe et al. (1998) investigated the influence of transcallosal inhibitory activity on functional brain asymmetry, employing three-dimensional magnetic resonance imaging for metabolic assessments. Their findings revealed metabolic alterations in the midbody of the corpus callosum and isthmus, which exhibited a negative correlation with activity in language-associated regions such as the left inferior cortex and the right superior temporal cortex. Specifically, as metabolic activity in the midbody of the corpus callosum increased, metabolic activity in these asymmetrically functioning cortical areas decreased. These observations suggest that interhemispheric inhibition, mediated through callosal fiber tracts, is intricately linked with the functional asymmetries observed between the left and right cortical regions. Such functional disparities serve to reinforce hemispheric lateralization or specialization in specific cognitive tasks, such as language processing predominantly governed by the left hemisphere.

Consequently, regulatory mechanisms between the left and right hemispheres facilitate a division of labor that optimizes foveal word recognition. This hemispheric partitioning enhances processing efficiency by mitigating superfluous interhemispheric inhibition. Such autonomous functioning of each hemisphere serves to preempt potential conflicts between the hemispheres, thereby streamlining cognitive processing.

In summary, the present investigation conducted two experiments to examine the mechanisms of foveal word recognition through the lens of hemispheric interactions. The findings revealed suboptimal performance in split word presentation compared to identical word presentation in the BVFs, implicating a division of labor across the hemispheres. This division appears to be driven by the need to circumvent inhibitory regulation that arises from simultaneous propagation of identical words to both hemispheres. By adhering to this hemispheric specialization, the bilateral processing of foveal words is consequently enhanced.

## Data availability statement

The original contributions presented in the study are included in the article/Supplementary materials, further inquiries can be directed to the corresponding author.

## Ethics statement

The studies involving humans were approved by Korea University Institutional Review Board (KUIRB-2021-0427-01). The studies were conducted in accordance with the local legislation and institutional requirements. The participants provided their written informed consent to participate in this study.

## Author contributions

SK: Conceptualization, Formal analysis, Funding acquisition, Investigation, Methodology, Project administration, Visualization, Writing – original draft, Writing – review & editing. KN: Supervision, Writing – review & editing.
